# Accessibility of Pulmonary Rehabilitation in the US

**DOI:** 10.1001/jamanetworkopen.2023.54867

**Published:** 2024-02-05

**Authors:** Peter A. Kahn, Walter S. Mathis

**Affiliations:** 1 Section of Pulmonary, Critical Care and Sleep Medicine, Yale School of Medicine, New Haven, Connecticut; 2 Department of Psychiatry, Yale School of Medicine, New Haven, Connecticut

## Abstract

This cross-sectional study examines nationwide travel time to pulmonary rehabilitation (PR) programs and PR access in the US.

## Introduction

Pulmonary rehabilitation (PR) is a multidisciplinary program combining exercise education and behavioral strategies to improve respiratory disease management and is an essential component of comprehensive care for patients with chronic respiratory diseases. PR has demonstrated meaningful benefits in improving exercise capacity, reducing the severity of dyspnea, and enhancing health-related quality of life, particularly in patients with chronic obstructive pulmonary disease, bronchiectasis, interstitial lung diseases, and pulmonary hypertension.^1,2^ Despite its proven efficacy, access to PR services remains a substantial issue, particularly for patients residing in rural areas. We therefore sought to understand nationwide travel time to PR programs as a marker for PR access.

## Methods

This cross-sectional study was not submitted for institutional review board approval and did not require informed consent because it used aggregate and deidentified public data and was not human participant research as defined by 45 CFR §46. We followed the STROBE reporting guideline.

Locations of PR sites were obtained from the livebetter.org website.^3^ Population and race and ethnicity data came from the American Community Survey 2021 five-year estimates, and urbanicity data came from the US Census Urban Areas designation. Minimum travel time to PR site was computed using an open source routing machine server as outlined in prior work.^4^ Travel times were computed from the center of every census block to the 25 closest PR sites, recording the minimum time. From these times, a population-weighted mean was computed for each census tract. We limited our analysis to the lower 48 states and Washington, DC.

We used a descriptive analysis approach, focusing on summarizing and interpreting the data in its existing form without adjusting for other variables or covariates. Data analysis was performed using R version 4.2.1 (R Project for Statistical Computing).

## Results

**Figure 1.**
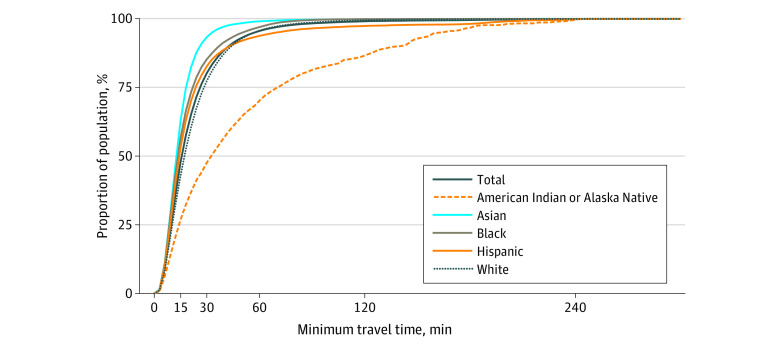
Cumulative Percentage of Population by Minimum Travel Time to Pulmonary Rehabilitation Facility by Race and Ethnicity

Using the US Census designation (total population living in the lower 48 states and Washington, DC, in the US: 327 534 982 people; 1 830 114 [0.6%] American Indian or Alaska Native, 17 974 172 [5.5%] Asian, 40 146 585 [12.3%] Black, 60 594 415 [18.5%] Hispanic, 195 265 295 [49.5%] White; 165 446 298 [50.5%] female; median age: 38.4 years), 1494 of 1759 PR sites (84.9%) were in urban areas. Densely populated urban areas and major cities offered the shortest travel times, with approximately 47.8% of the total US population living within a 15-minute drive of a PR program (Figure 1). Expanding this threshold to a 30-minute drive included an additional 32.5% of the population, primarily residing in suburban areas surrounding these urban centers.

**Figure 2.**
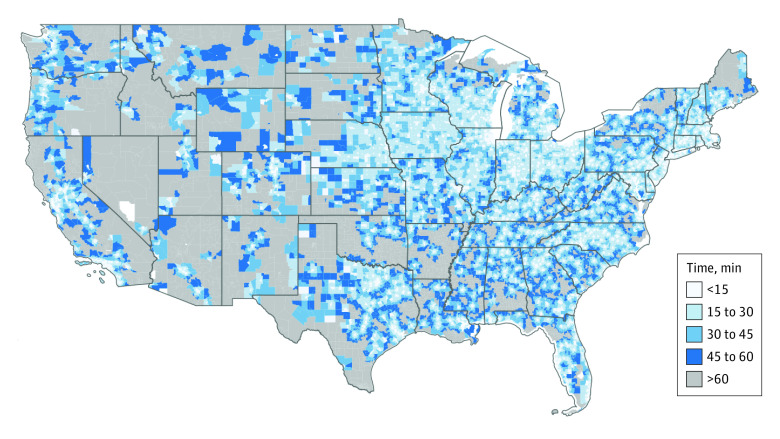
Population-Weighted Mean Minimum Travel Time to Pulmonary Rehabilitation Facilities by Census Tract

However, for individuals living in rural and sparsely populated regions, access to PR programs was limited. These areas, particularly in the western and midwestern parts of the country, often required travel times exceeding 30 minutes, with many regions (representing more than 14 000 000 people) necessitating more than a 1-hour drive to reach the nearest PR program (Figure 2).

Our data also found racial disparities in access to PR programs. While 26.9% of the American Indian and Alaska Native population lived within a 15-minute drive to a PR program, 29.7% lived more than 60 minutes away (Figure 1). This distribution differed from other race and ethnicity groups which skewed toward low access times.

## Discussion

Our study found substantial geographic disparities in access to PR programs across the US. By focusing on travel times instead of geodesic distance, our findings offer a more realistic depiction of the challenges faced by many individuals, particularly those in rural and sparsely populated regions and minoritized racial and ethnic groups.

Our study has limitations. While differential race and ethnicity of urban and rural areas of the US likely helps explain some of the unequal PR access patterns we observed, additional prospective analyses are required to definitively understand these disparities.^5^ Our study relied solely on travel time and does not account for cost, public transit routing, per capita availability, or patient-level factors such as walk distance or disease characteristics. Furthermore, the PR directory used does not contain information about program quality, availability, or schedules and was collected using a combination of public and voluntarily submitted data in 2023.

The disparities highlighted in our study underscore the need for innovative solutions to improve access to PR services for those in underserved areas. Many of these geographic disparities likely reflect systemic resource scarcity in the rural areas evaluated that extend beyond PR programs alone. While innovative solutions such as virtual PR can help bridge these gaps in the short term,^6^ long-term solutions will require collaboration between policy makers and those providing health care to those in underresourced areas.

## Notes

Supplement.
Data Sharing Statement
