# Supplementary material for: Accessibility of Pulmonary Rehabilitation in the US
Source: JAMA Netw Open. 2024 Feb 5;7(2):e2354867. doi: 10.1001/jamanetworkopen.2023.54867 (PMC10844999; doi:10.1001/jamanetworkopen.2023.54867)
Supplement: Supplement. — Data Sharing Statement [file jamanetwopen-e2354867-s001.pdf]

Data Sharing Statement

Kahn. Accessibility of Pulmonary Rehabilitation in the US. *JAMA Netw Open*. Published February 05, 2024. doi:10.1001/jamanetworkopen.2023.54867

Data
Data available: No
